# Supplementary material for: Phenotypic selection through cell death: stochastic modelling of O-6-methylguanine-DNA methyltransferase dynamics
Source: R Soc Open Sci. 2020 Jul 8;7(7):191243. doi: 10.1098/rsos.191243 (PMC7428254; doi:10.1098/rsos.191243)
Supplement: Supplemental.pdf [file rsos191243supp1.pdf]

SUPPLEMENTAL MATERIAL

Phenotypic selection through cell death: stochastic modelling of O-6-methylguanine-DNA methyltransferase dynamics

Ayoub Lasri[1*], Viktorija Juric[1], Maité Verreault[2], Franck Bielle[3], Ahmed Idbaih[3], Alexander Kel[4,5], Brona Murphy[1] and Marc Sturrock[1]

[*]Correspondence: lasriay@gmail.com
[1]Department of Physiology and Medical Physics, Royal College of Surgeons in Ireland, York House, Dublin, Ireland
Full list of author information is available at the end of the article

S1 Accounting for active degradation of MGMT protein

We first assumed MGMT was stable and therefore neglected to include an active degradation term. However, *Smalley et al.* [1] reported that the half life of MGMT was about 60 hours. We therefore also explored the impact of accounting for active MGMT protein degradation in our model. In particular, we included the reactions

$$MGMT \xrightarrow{\mathrm{d}_2} \varnothing \tag{S1}$$

and

$$REF \xrightarrow{\mathrm{d}_2} \varnothing \tag{S2}$$

where d_2 is the protein degradation rate. As seen in Figure S1, this had no impact on either the phenotypic selection or cell viability recovery for a large range of degradation rates. Furthermore, in order to study the impact of degradation in a more systematic manner, we investigated varying the protein degradation rates while matching the mean protein level to their baseline levels prior to TMZ administration without degradation (using parameters in Table 1). To achieve this we increased the baseline translation rate using the following formula derived from mean field steady state equations

$$b_2^* = \frac{b_2(d_2 + \mu)}{\mu} \tag{S3}$$

Here we again found no appreciable change in our results for a broad range of protein noise levels. Altogether, the results in this section show that our model is robust to accounting for MGMT protein degradation.

S2 Accounting for suicide enzymatic activity of MGMT

To account for the suicide enzymatic activity of MGMT [2], we explored the effect of including an additional reaction to MGMT only (excluding the reference protein). The rate of this degradation has to match the de-alkylation activity. Hence, we

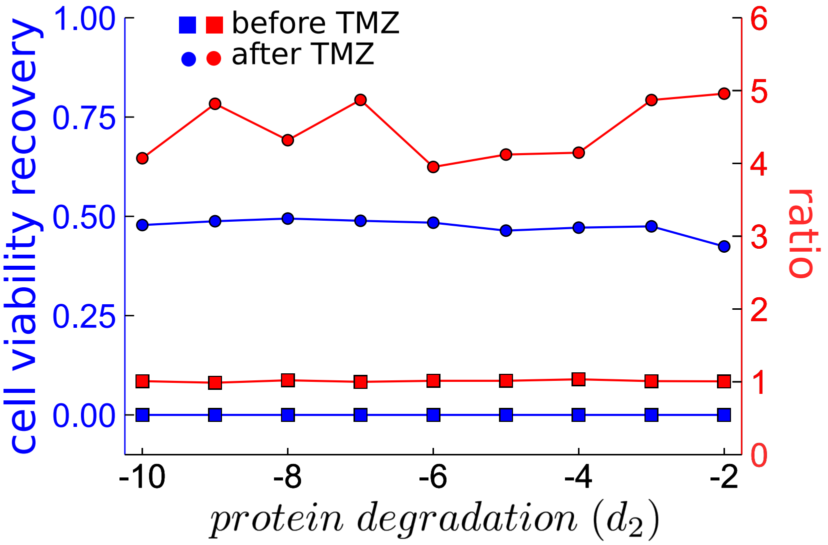

Supplemental Material, Figure S1 Plot shows cell viability recovery and ratio defined by $\frac{<MGMT>}{<REF>}$ computed from simulations of model defined by chemical reactions (2.10), (2.12)-(2.14), (2.17), (2.18) and (2.28), (S1)-(S2) and ODE solution (2.4) in absence of TMZ or a time discretisation of (2.1) and (2.3) in the presence of TMZ, using parameters defined in Table 1 (unless otherwise stated) and initial conditions in Table S1 in response to TMZ $= 800\ \mu M$ using f_4 with $t_{in} = 144$ hours. Plot shows how degradation rates d_2 are related to phenotypic selection (ratio) and cell viability recovery before (140 hours) and following TMZ administration (288 hours).

include an additional reaction for MGMT:

$$MGMT_i \xrightarrow{\mathrm{sDNA_{DAM_i}}} \varnothing \tag{S4}$$

and apply the law of mass action to it and reactions (2.13), (2.19) to arrive at the mean field ordinary differential equation for MGMT in cell i

$$\frac{dMGMT_i}{dt} = b_2 \cdot mRNA\_MGMT_i - d_2 \cdot MGMT_i - s \cdot MGMT_i \cdot DNA_{DAM_i}. \tag{S5}$$

The final term on the right hand side corresponds to the de-alkylation rate. If we substitute in the steady state term for DNA_{DAM} defined in (2.25) to this term we arrive at the MGMT degradation rate associated with MGMT enzymatic suicide activity:

$$s \cdot MGMT_i \cdot DNA^*_{DAM_i} = sMGMT_i \frac{k_d \cdot TMZ}{k_d \cdot TMZ + s \cdot MGMT_i}, \tag{S6}$$

which after rearranging becomes

$$s \cdot MGMT_i \cdot DNA^*_{DAM_i} = \frac{k_d \cdot TMZ}{(k_d \cdot TMZ)/(s \cdot MGMT_i) + 1}. \tag{S7}$$

We can check the limits of this term to make sure it makes sense.

$$\lim_{s \to 0} \frac{k_d \cdot TMZ}{(k_d \cdot TMZ)/(s \cdot MGMT_i) + 1} = 0, \tag{S8}$$

This limit shows that if $s = 0$ there is no de-alkylation and therefore no MGMT suicide activity.

$$\lim_{s \to \infty} \frac{k_d \cdot TMZ}{(k_d \cdot TMZ)/(s \cdot MGMT_i) + 1} = k_d \cdot TMZ, \tag{S9}$$

In the limit of instantaneous de-alkylation, i.e. $s \to \infty$ we find there is still some suicide activity of MGMT as the MGMT still must be consumed to perform its function.

$$\lim_{TMZ \to 0} \frac{k_d \cdot TMZ}{(k_d \cdot TMZ)/(s \cdot MGMT_i) + 1} = 0, \tag{S10}$$

If $TMZ = 0$ there is no death and therefore no MGMT suicide activity. Finally, if TMZ concentration is very high, i.e. $TMZ \to \infty$ then we see the suicide activity is proportional to the amount of MGMT, as reflected in the following limit

$$\lim_{TMZ \to \infty} \frac{k_d \cdot TMZ}{(k_d \cdot TMZ)/(s \cdot MGMT_i) + 1} = s \cdot MGMT. \tag{S11}$$

Remarkably, even with this biased degradation term only impacting MGMT, we still observe phenotypic selection and cell viability recovery (Figure S2 (A)). However, we note that the cell viability recovery is diminished. We found that by adjusting the TMZ mediated cell death rate k_d, not only we could recapitulate the cell viability recovery reported in the main paper, (compare Figure 2(D) with Figure S2(B)), but in fact we could enhance it (Figure S2(C)). Hence, omitting or accounting for MGMT suicide activity and MGMT degradation does not affect the ability of our model to exhibit phenotypic selection.

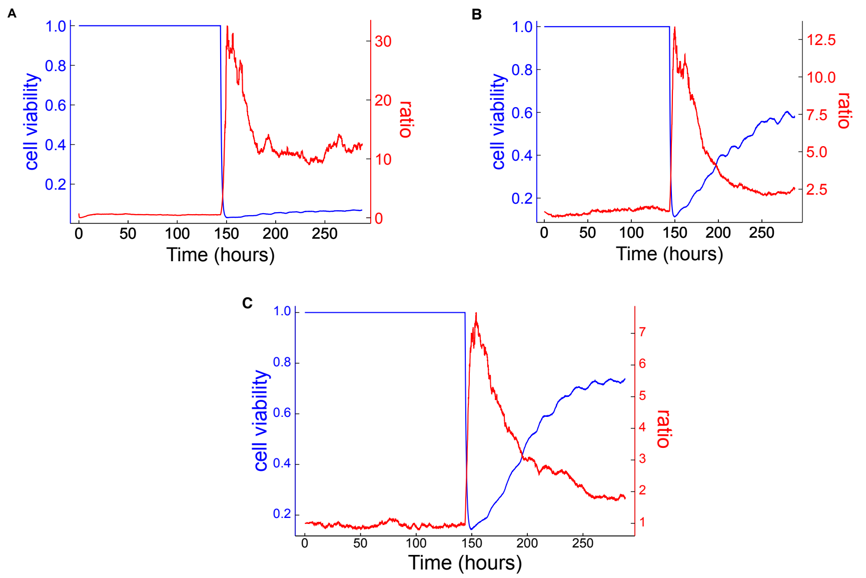

Supplemental Material, Figure S2 Plots show cell viability recovery and ratio defined by $\frac{<MGMT>}{<REF>}$ computed from simulations of model defined by chemical reactions (2.10), (2.12)-(2.14), (2.17), (2.18) and (2.28), (S1)-(S2) and ODE solution (2.4) in absence of TMZ or a time discretisation of (2.1) and (2.3) in the presence of TMZ, using parameters defined in Table 1 (unless otherwise stated) and initial conditions in Table S1 in response to TMZ $= 800\ \mu M$ using f_4 with $t_{in} =$ 144 hours. Plot **(A)** shows results for k_d as in Table 1. Plot **(B)** shows results for $k_d = 0.024\ \mu M\ h^{-1}$. Plot **(C)** shows results for $k_d = 0.0024\ \mu M\ h^{-1}$.

Table of initial conditions used in simulations

Table S1 The table below summarises the initial values used.

Initial condition	Value	Units
MGMT mRNA	10	Copy number
MGMT	100	Copy number
REF mRNA	10	Copy number
REF	100	Copy number
$[TMZ]$	0.0	μM

Parameter sets that yield phenotypic selection for general model

Posterior parameter distributions plot showing the parameter distributions (plotted on a logarithmic scale) that fit the synthetic phenotypic selection data defined in methods section ($Error_1$ and $Error_2$) using model defined by chemical reactions (2.10), (2.12)-(2.14), (2.17), (2.18) and (2.28) and ODE solution (2.4) in absence of TMZ or a time discretisation of (2.1) and (2.3) in the presence of TMZ, using initial conditions in Table S1.

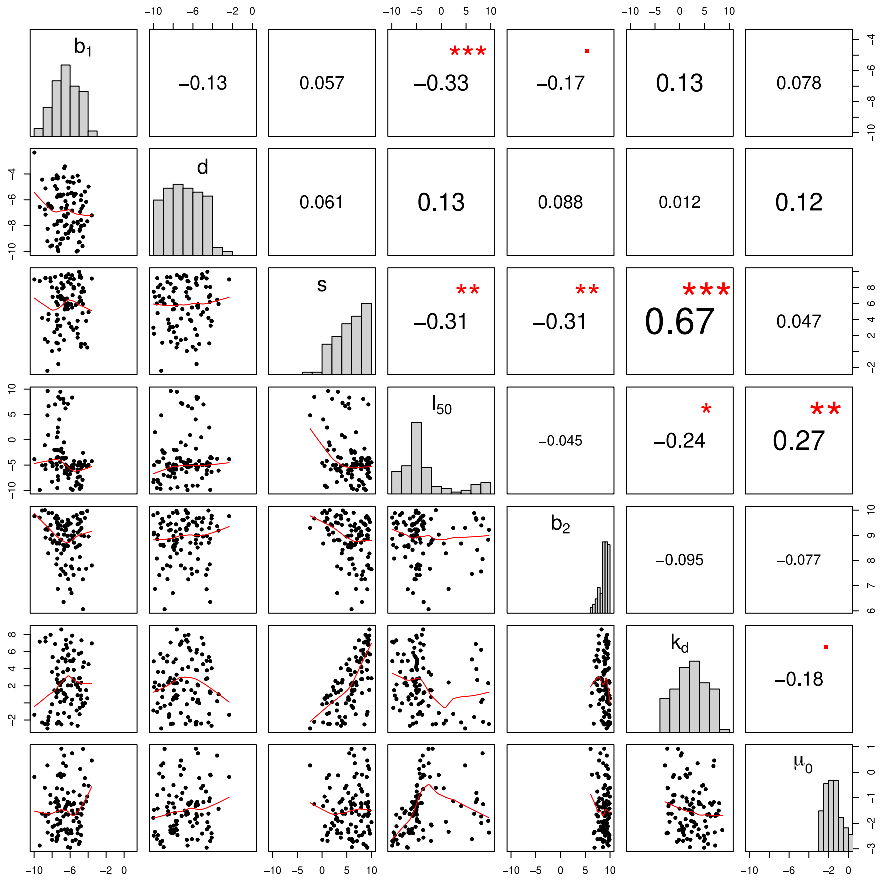

Supplemental Material, Figure S3 Parameter sets that yield phenotypic selection and cell viability recovery. General model. Limits on x-axes correspond to ranges of Uniform prior distributions used to initialise ABC inference. The diagonal plots show the posterior distributions. Lower triangular plots show scatter plots of parameter distributions with lowess smoothed line overlaid. Upper triangular plots show Pearson correlation with a point representing a p-value less than 0.05, an asterisk representing a p-value less than 0.01, two asterisks representing a p-value less than 1e-3 and three asterisks representing a p-value less than 1e-4.

Parameter sensitivity analysis for general model

Sensitivity analysis of parameter sets that yield phenotypic selection in the case of the general model (Figure S3).

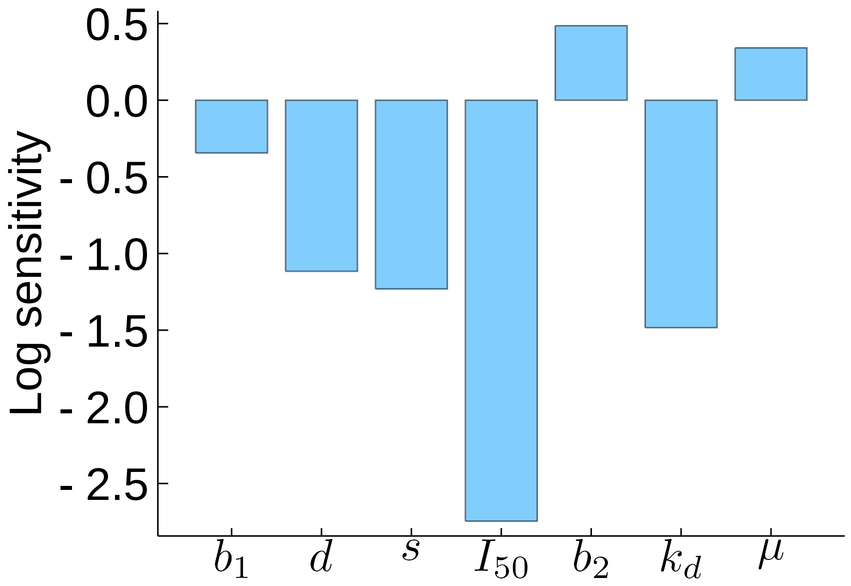

Supplemental Material, Figure S4 Parameter sensitivity analysis for the general model. Sensitivity of parameters for non-data constrained reduced model as computed by inverting the covariance matrix of the final probability distribution shown on the diagonal of Figure S3.

Parameter sets that yield phenotypic selection for data constrained model

Posterior parameter distributions plot showing the parameter distributions that fit the synthetic phenotypic selection data with cell viability data and fixed cell growth rate. Specifically we used the error functions $Error_1$, $Error_2$ and $Error_3$ defined in the methods section and use the model defined by chemical reactions (2.10), (2.12)-(2.14), (2.17), (2.18) and (2.27) and ODE solution (2.4) in absence of TMZ or a time discretisation of (2.1) and (2.3) in the presence of TMZ, using initial conditions in Table S1.

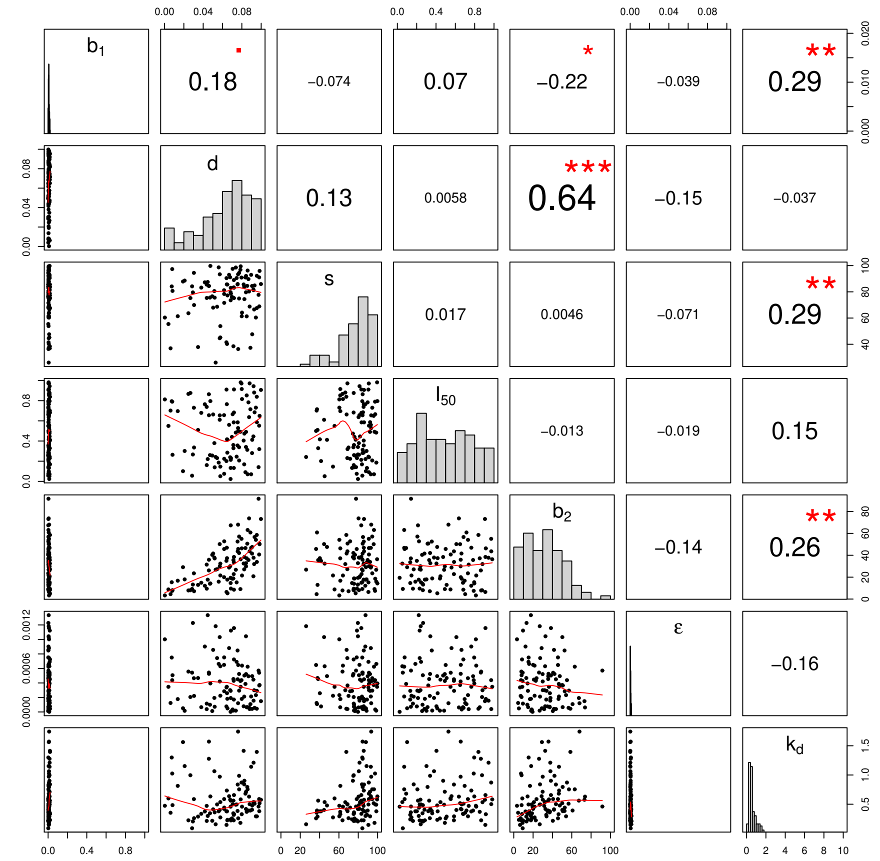

Supplemental Material, Figure S5 Parameter sets that yield phenotypic selection. Data-constrained model. Limits on x-axes correspond to ranges of Uniform prior distributions used to initialise ABC inference. The diagonal plots show the posterior distributions. Lower triangular plots show scatter plots of parameter distributions with lowess smoothed line overlaid. Upper triangular plots show Pearson correlation with a point representing a p-value less than 0.05, an asterisk representing a p-value less than 0.01, two asterisks representing a p-value less than 1e-3 and three asterisks representing a p-value less than 1e-4.

Parameter sensitivity analysis for data constrained model

Sensitivity analysis of parameter sets that yield phenotypic selection in the case of the data constrained model (Figure S5).

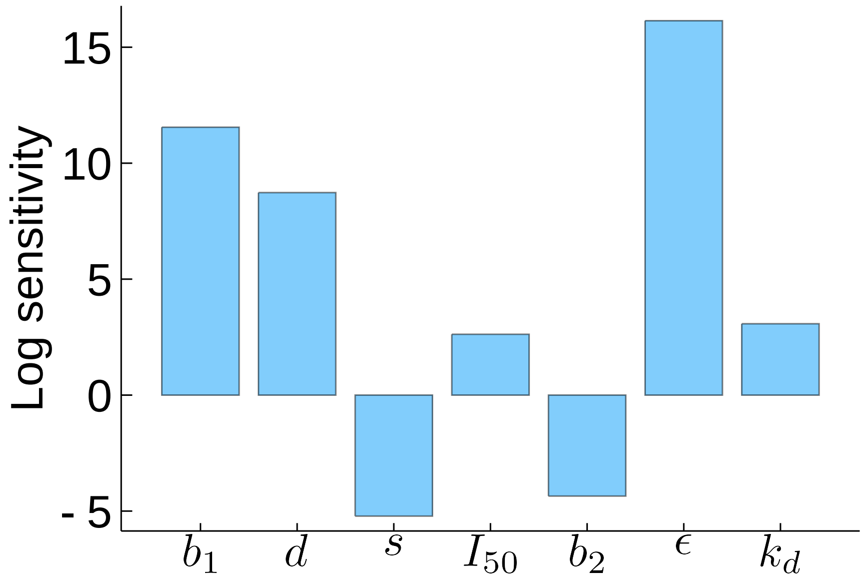

Supplemental Material, Figure S6 Parameter sensitivity analysis for data-constrained model. Sensitivity of parameters for model fitted to cell viability experiment as computed by inverting the covariance matrix of the final probability distribution shown on the diagonal of Figure S5.

De-alkylation rate and TMZ mediated cell death relation to phenotypic selection and cell viability recovery

In this section, we present results corresponding to varying the TMZ mediated cell death parameter k_d while keeping the rest of the parameters in Table 1 constant (Figure S7(A)). We found that by making k_d small, phenotypic selection and cell viability recovery were lost, implying that cell death is crucial for investigating both phenotypic selection and cell viability recovery. We also investigated how cell viability and phenotypic selection are related to the de-alkylation rate parameter (s) in the general and the full model without the steady state approximation explained in the methods section (Figure S7(B)). We found that for high values of s, cell viability recovery is maximised and phenotypic selection is minimised, we note though that both models had similar behaviour for large values of s. While decreasing the de-alkylation rate, cell viability recovery was lost and phenotypic selection reached higher values. We noticed that the models behaviour diverges for small values of s implying that the steady state approximation is only valid for higher values of s. We note that the values of s which produced phenotypic selection were very large, therefore we expect our steady state approximation to be valid.

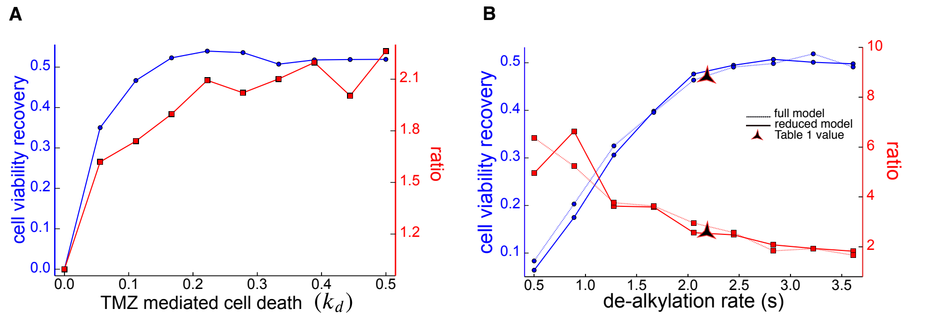

Supplemental Material, Figure S7 De-alkylation rate and TMZ mediated cell death relation to phenotypic selection and cell viability recovery. Plots show cell viability recovery and ratio defined by $\frac{<MGMT>}{<REF>}$ computed from simulations of model defined by chemical reactions (2.10), (2.12)-(2.14), (2.17), (2.18) and (2.28), (S1)-(S2) and ODE solution (2.4) in absence of TMZ or a time discretisation of (2.1) and (2.3) in the presence of TMZ, using parameters defined in Table 1 (unless otherwise stated) and initial conditions in Table S1 in response to TMZ $= 800\ \mu M$ using f_4 with t_{in} $=$ 144 hours. Plot **(A)** shows how varying the TMZ mediated cell death (k_d) parameter affects phenotypic selection and cell viability recovery. Plot **(B)** shows how the de-alkylation rate s (in base 10 logarithmic scale) is related to phenotypic selection and cell viability recovery. The solid line (resp. dotted line) corresponds to simulations produced by the model defined by chemical reactions (2.10), (2.12)-(2.14), (2.17), (2.18) and (2.28) (resp. (2.8)-(2.18)).

Cells response to TMZ: the case where $s = 0$

Finally we present the special case of simulating the model defined by chemical reactions (2.10), (2.12)-(2.14), (2.17), (2.18) and (2.27) and ODE solution (2.4) in absence of TMZ or a time discretization of (2.1) and (2.3) in the presence of TMZ, using initial conditions in Table S1 where the de-alkylation rate is set to zero and all other parameters are as defined in Table 1. This shows that the MGMT is indeed providing protection to the cells in the presence of TMZ and without any dealkylation the cells quickly die and there is no difference in behaviour between MGMT and the reference protein.

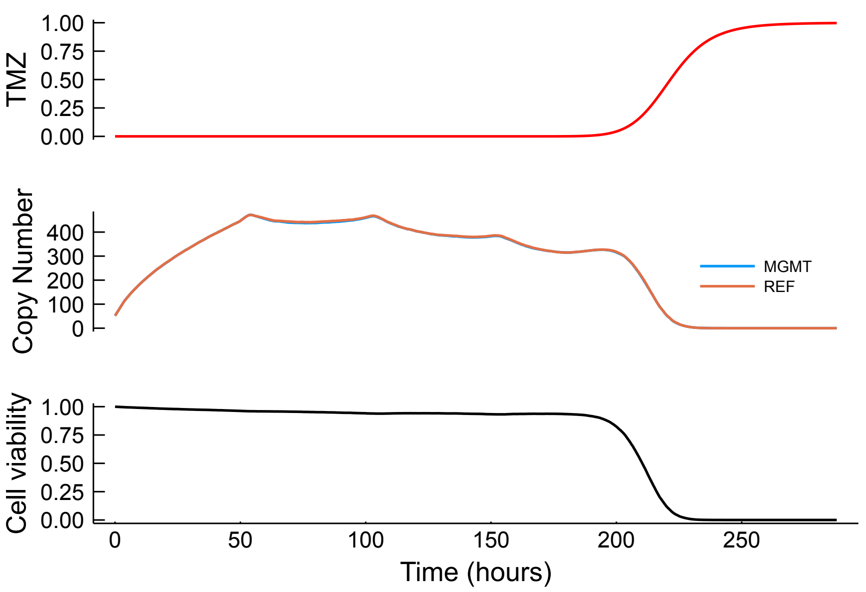

Supplemental Material, Figure S8 Cells response to TMZ: the case where MGMT is not acting as a de-alkylating agent, $s = 0$. Plots show TMZ dosage, proteins copy number and cell viability simulations in response to TMZ $= 800\ \mu M$ following f_3, from model defined by chemical reactions (2.10), (2.12)-(2.14), (2.17), (2.18) and (2.27) and ODE solution (2.4) in absence of TMZ or a time discretisation of (2.1) and (2.3) in the presence of TMZ, using parameters defined in Table 2 and initial conditions in Table S1. Post-TMZ administration, all the cells died and no phenotypic selection is observed.

Author details
[1]Department of Physiology and Medical Physics, Royal College of Surgeons in Ireland, York House, Dublin, Ireland. [2]Inserm U 1127, CNRS UMR 7225, Sorbonne Université, Institut du Cerveau et de la Moelle épinière, ICM, F-75013, Paris, France. [3]Sorbonne Université, Inserm, CNRS, UMR S 1127, Institut du Cerveau et de la Moelle épinière, ICM, AP-HP, Hôpitaux Universitaires La Pitié Salpêtrière - Charles Foix, Service de Neurologie 2-Mazarin, F-75013, Paris, France. [4]Department of Research and Development, geneXplain GmbH, Wolfenbüttel D-38302, Germany. [5]Laboratory of Pharmacogenomics, Institute of Chemical Biology and Fundamental Medicine, Novosibirsk 630090, Russia.

References

1. Smalley, S., Chalmers, A.J., Morley, S.J.: mTOR inhibition and levels of the DNA repair protein MGMT in T98g glioblastoma cells. Molecular Cancer **13**(1), 144 (2014). doi:10.1186/1476-4598-13-144
2. Christmann, M., Verbeek, B., Roos, W.P., Kaina, B.: O6-Methylguanine-DNA methyltransferase (MGMT) in normal tissues and tumors: Enzyme activity, promoter methylation and immunohistochemistry. Biochimica et Biophysica Acta (BBA) - Reviews on Cancer **1816**(2), 179–190 (2011). doi:10.1016/j.bbcan.2011.06.002

Additional files

Additional file 1 — Experimental data: cell growth.
(xlsx 12 KB).

Additional file 2 — Experimental data: cell viability assay.
(xlsx 12 KB).
